# Assessment of CO₂ sequestration potential in the Abu Sannan oil field for sustainable energy and environment using seismic modeling, Western Desert, Egypt

**DOI:** 10.1038/s41598-025-28731-x

**Published:** 2025-12-05

**Authors:** Mohammed Amer, Walid M. Mabrouk, Amr M. Eid, Ahmed M. Noureldin, Ahmed Metwally

**Affiliations:** Geophysics Department, Faculty of Science, Cairo University, Giza, 12613 Egypt

**Keywords:** Carbon capture and storage, Abu Sannan oil field, Seismic modeling, Sustainability, Abu Roash D, Western desert, Energy science and technology, Solid Earth sciences

## Abstract

Global efforts to mitigate climate change have highlighted Carbon Capture and Storage (CCS) as a practical solution for reducing atmospheric CO₂, with depleted oil and gas reservoirs (DOGR) being particularly attractive due to their well-characterized structures, proven sealing capacity, and existing infrastructure. Egypt’s Western Desert, with its extensive petroleum history and mature fields, presents significant potential for large-scale CCS deployment. This study investigates the CO₂ sequestration potential of the Abu Sannan Oil Field, focusing on the Abu Roash D reservoir. Structural interpretation of seismic data identified a system of normal faults forming tilted blocks, horsts, and grabens, while petrophysical analysis from well logs assessed key properties such as porosity, shale content, water saturation, and permeability. A three-dimensional (3D) geological model integrating seismic interpretation, well logs, and petrophysical parameters was developed to estimate storage capacity. Results indicate that the southeastern closure, representing the highest structural part of the field, is the most promising site, with excellent porosity (~ 25%), very low shale content (~ 5%), moderate water saturation (~ 40%), and relatively uniform permeability, ensuring high injectivity, storage efficiency, and reservoir homogeneity. The 3D model estimates that Abu Roash D can securely accommodate approximately 72,657 Mt of CO₂, with the Khoman Formation providing long-term containment integrity. The reservoir’s combination of favorable structural geometry, high-quality petrophysical properties, and existing infrastructure makes it a prime candidate for large-scale CCS, supporting Egypt’s decarbonization strategy and contributing to global climate mitigation initiatives.

## Introduction

 Global warming, primarily driven by the excessive release of greenhouse gases such as carbon dioxide (CO₂), has become a critical global concern. Rising temperatures are contributing to widespread environmental degradation, including sea level rise, melting polar ice, more frequent extreme weather events, and disruptions to both natural ecosystems and human livelihoods. Addressing this challenge requires urgent and coordinated global action to reduce CO₂ emissions and mitigate the long-term impacts of climate change. A variety of strategies have been proposed to combat global warming, including the adoption of renewable energy sources, improvements in energy efficiency, and the implementation of carbon management technologies^1,2^.

**Fig. 1:**
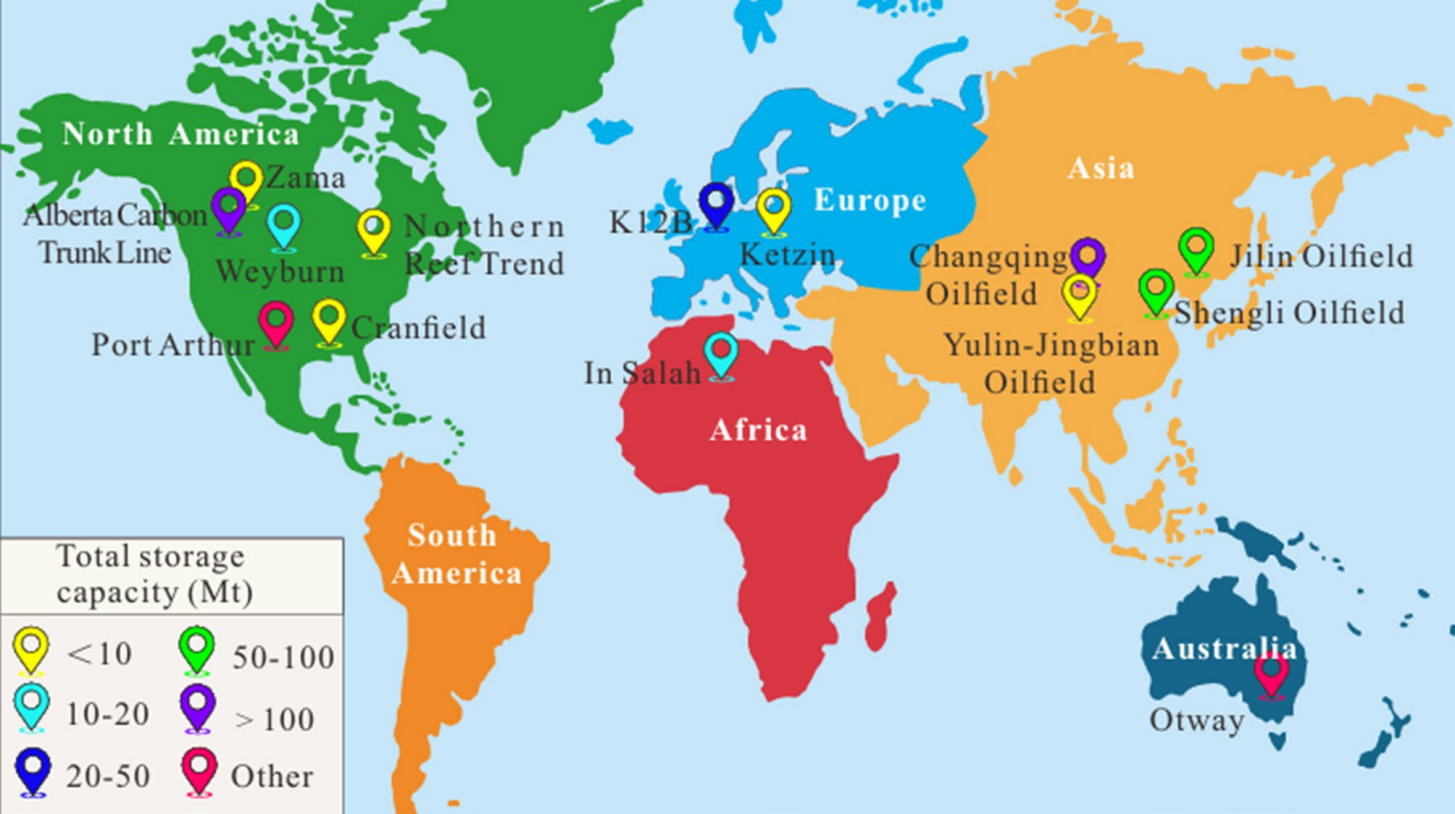
Global CO₂ storage projects utilizing DOGRs, which are characterized by reservoirs that are no longer economically viable for hydrocarbon production^5^.

Among these solutions, Carbon Capture and Storage (CCS) has gained significant attention as a practical and scalable method to reduce atmospheric CO₂ levels. Recognizing the urgency of climate action, major economies such as the European Union, the United States, Japan, South Korea, and China have committed to achieving carbon neutrality within the coming decades, with China specifically aiming to peak emissions before 2030 and reach carbon neutrality by 2060. Figure 1 presents global CO₂ storage projects implemented exclusively in depleted oil and gas reservoirs (DOGRs). These ambitious goals underscore the importance of CO₂ storage initiatives worldwide. Geological storage options for CO₂ include deep saline aquifers, non-minable coal seams, subsea formations, and notably, DOGR. Geological formations offer varying potentials for long-term CO₂ storage, each with distinct advantages and limitations. Deep saline aquifers represent the largest theoretical capacity, estimated at several hundred to several thousand gigatons (Gt) of CO₂. However, this potential is accompanied by considerable uncertainty due to limited characterization and heterogeneity of the reservoirs. Non-minable coal seams provide comparatively smaller capacities (tens to hundreds of Gt) and face technical challenges related to CO₂ adsorption and desorption dynamics. Subsea formations may contribute regional-scale capacities on the order of hundreds of Gt, but their development is constrained by elevated costs and complex monitoring requirements.

While previous studies have reviewed CCS technologies broadly, there is a lack of detailed analysis focusing specifically on real-world DOGR projects, the key factors influencing their success, and the mechanisms by which CO₂ is securely trapped. Advancing this understanding is essential to support global climate goals and enhance the role of CCS in enabling a sustainable, low-carbon future^3,4^.

The process of CO₂ storage in DOGR involves several critical stages, beginning with the capture of CO₂ from industrial or energy-related sources. Once captured, the CO₂ undergoes dehydration and compression to ensure it meets the required physical conditions for underground injection, typically in either liquid or supercritical form to maximize storage efficiency. Notably, the equipment and operational techniques used for CO₂-enhanced oil and gas recovery are largely applicable to CO₂ storage, allowing for cost-effective repurposing of existing infrastructure. The storage process generally includes CO₂ capture, gas treatment and separation (such as removal of water and impurities), compression, and injection into the reservoir using methods like miscible or immiscible flooding^5^. After injection, long-term storage integrity is ensured through continuous monitoring. These technical steps have been widely documented in previous research, and as such, this paper will not revisit those operational details to maintain a concise focus on other key aspects of CO₂ storage in DOGR^6,7^.

Egypt, as a leading energy producer with vast natural resources, faces the strategic imperative of balancing economic growth with environmental responsibility. In response to the global call for climate action, Egypt has identified CCS as a key pillar in its national strategy to reduce carbon emissions and promote sustainable development. The Western Desert, in particular, represents a highly promising region for CCS deployment due to its extensive hydrocarbon reserves, well-established oil and gas infrastructure, and favorable geological characteristics. This region hosts a dense network of pipelines, production facilities, and depleted oil and gas reservoirs, making it exceptionally well-suited for the integration of CCS technologies. Given Egypt’s continued reliance on fossil fuels, the implementation of CCS in the Western Desert offers a practical and immediate pathway to reduce CO₂ emissions while maintaining energy security. Moreover, this approach aligns with Egypt’s broader commitment to environmental stewardship and its active role in supporting global decarbonization goals^8,9^.

On a global scale, CCS has emerged as a critical technology in efforts to mitigate greenhouse gas emissions, particularly from industrial and energy-intensive sectors. Among various geological storage options, DOGR have gained prominence due to their proven containment capabilities, extensive storage potential, and the availability of existing infrastructure advantages that are abundantly present in Egypt’s Western Desert. The successful deployment of CCS in such settings relies heavily on advanced three-dimensional (3D) geological modeling, which integrates data from well logs, seismic surveys, and core analyses to accurately characterize subsurface conditions.

Seismic modeling has long been a cornerstone of hydrocarbon exploration, primarily employed to define reservoir architecture, identify structural and stratigraphic traps, and monitor hydrocarbon migration during production. In CCS projects, however, its application is directed toward assessing storage capacity, verifying caprock integrity, and monitoring the migration and long-term stability of injected CO₂. While both contexts rely on precise subsurface imaging and characterization, their objectives diverge: hydrocarbon exploration is focused on maximizing resource recovery, whereas CCS emphasizes secure storage and environmental safety over extended timescales. This contrast highlights the adaptability of seismic modeling and underscores its importance as both a mature exploration tool and a critical technique in advancing CCS research. In this study, 3D seismic modeling was carried out through a structured workflow. The process began with seismic interpretation to identify and track key horizons and faults across the survey area. These interpretations were then used to construct structural contour maps that define the geometry of the subsurface units. The mapped horizons and fault networks provided the framework for building a 3D static model of the reservoir and caprock system. These models facilitate detailed assessment of structural features, reservoir properties, and sealing mechanisms, while also incorporating simulation techniques to account for heterogeneity and predict CO₂ migration behavior. By leveraging the Western Desert’s mature oil and gas infrastructure alongside high-resolution 3D modeling, Egypt is uniquely positioned to implement cost-effective, large-scale CCS projects. This integration not only enhances the viability of long-term CO₂ storage but also supports Egypt’s transition toward a more sustainable energy future, reinforcing its leadership in regional climate mitigation efforts^4,10^.

Building on Egypt’s strategic potential for CCS in the Western Desert, this study specifically investigates the Abu Sannan Oil Field as a candidate site for CO₂ sequestration. Located within a region that benefits from extensive oil and gas infrastructure and well-documented geological formations, the field presents favorable conditions for implementing CCS. Particular attention is given to the fractured carbonate of the Abu Roash D Formation, a key stratigraphic unit that has historically served as a hydrocarbon reservoir and now offers promising characteristics for permanent CO₂ storage. The primary objective of this research is to develop a high-resolution 3D static geological model of the Abu Sannan Oil Field to assess its CO₂ storage capacity and suitability. By integrating seismic reflection data with advanced reservoir modeling techniques, the study aims to address critical factors such as reservoir continuity, structural complexity, and sealing efficiency. The results will provide essential insights into the feasibility of using depleted reservoirs in the Western Desert for safe and long-term CO₂ storage, thereby supporting Egypt’s broader efforts to reduce emissions and transition toward sustainable energy practices^11–13^.

## Geological setting

Egypt’s Western Desert is one of the country’s most prolific hydrocarbon provinces and forms part of the larger North African sedimentary basin system. It encompasses several well-established petroleum basins, including Abu Gharadig, Bahariya, Matruh, and Alamein, all of which have been the focus of extensive exploration and production activities for decades. These basins are characterized by a thick sedimentary succession, favorable structural traps, and mature petroleum systems, with hydrocarbon production primarily sourced from Jurassic to Upper Cretaceous reservoirs. In addition to its petroleum significance, the Western Desert is increasingly being considered for CCS due to the availability of depleted reservoirs, comprehensive geological data, and an extensive network of pipelines and production infrastructure that can be repurposed for CO₂ sequestration^14–17^.

**Fig. 2:**
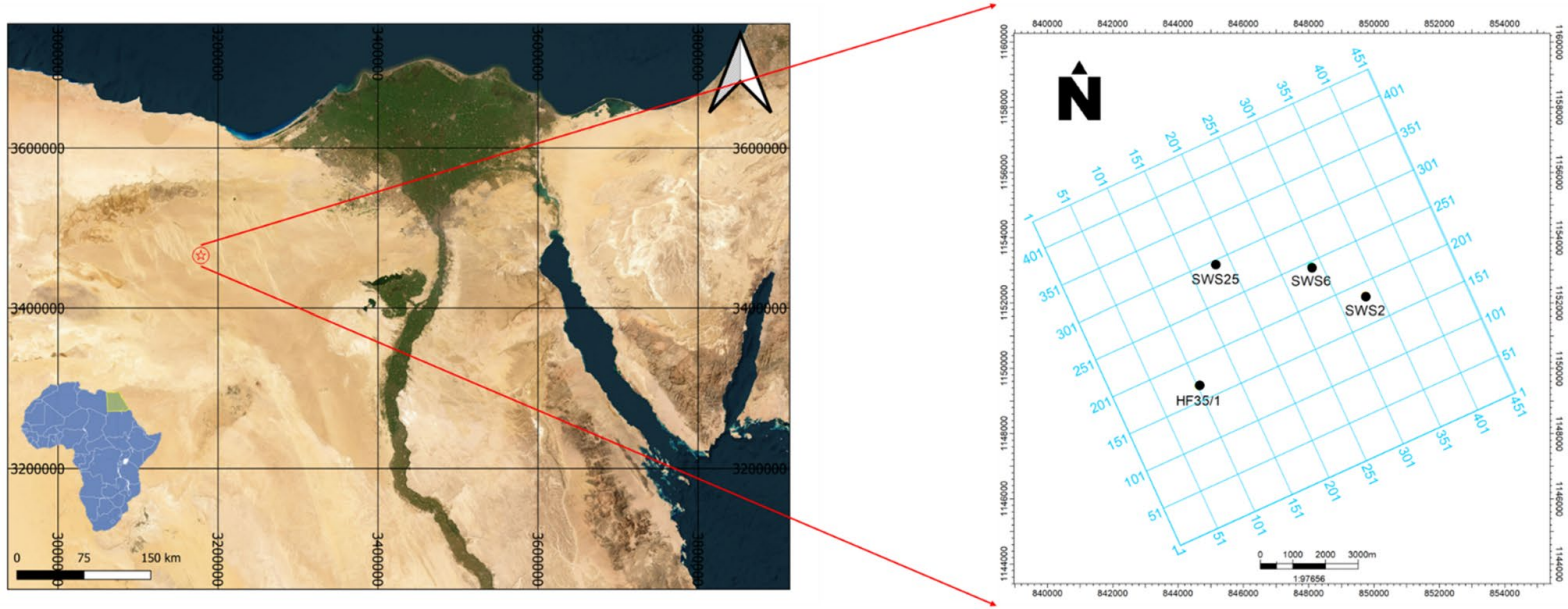
Map showing the location of the study area, seismic survey coverage, and the spatial distribution of wells within the Abu Sannan Field^28^ (Map created using QGIS, Version 3.28, https://qgis.org ).

The Abu Sannan Oil Field is located within the Abu Gharadig Basin in the northern part of the Western Desert, approximately 200 km southwest of Alexandria (Fig. 2). This field has been a significant contributor to Egypt’s oil production, primarily targeting reservoirs within the Cretaceous succession. Following years of production, many of the field’s reservoirs are now depleted or nearing the end of their productive life, making them viable candidates for CO₂ storage. The field’s strategic location, coupled with its proximity to potential CO₂ sources and existing infrastructure, supports its suitability for geological sequestration. In particular, the availability of high-resolution seismic data and detailed well logs from extensive exploration efforts enables a thorough evaluation of its storage potential^18,19^.

**Fig. 3:**
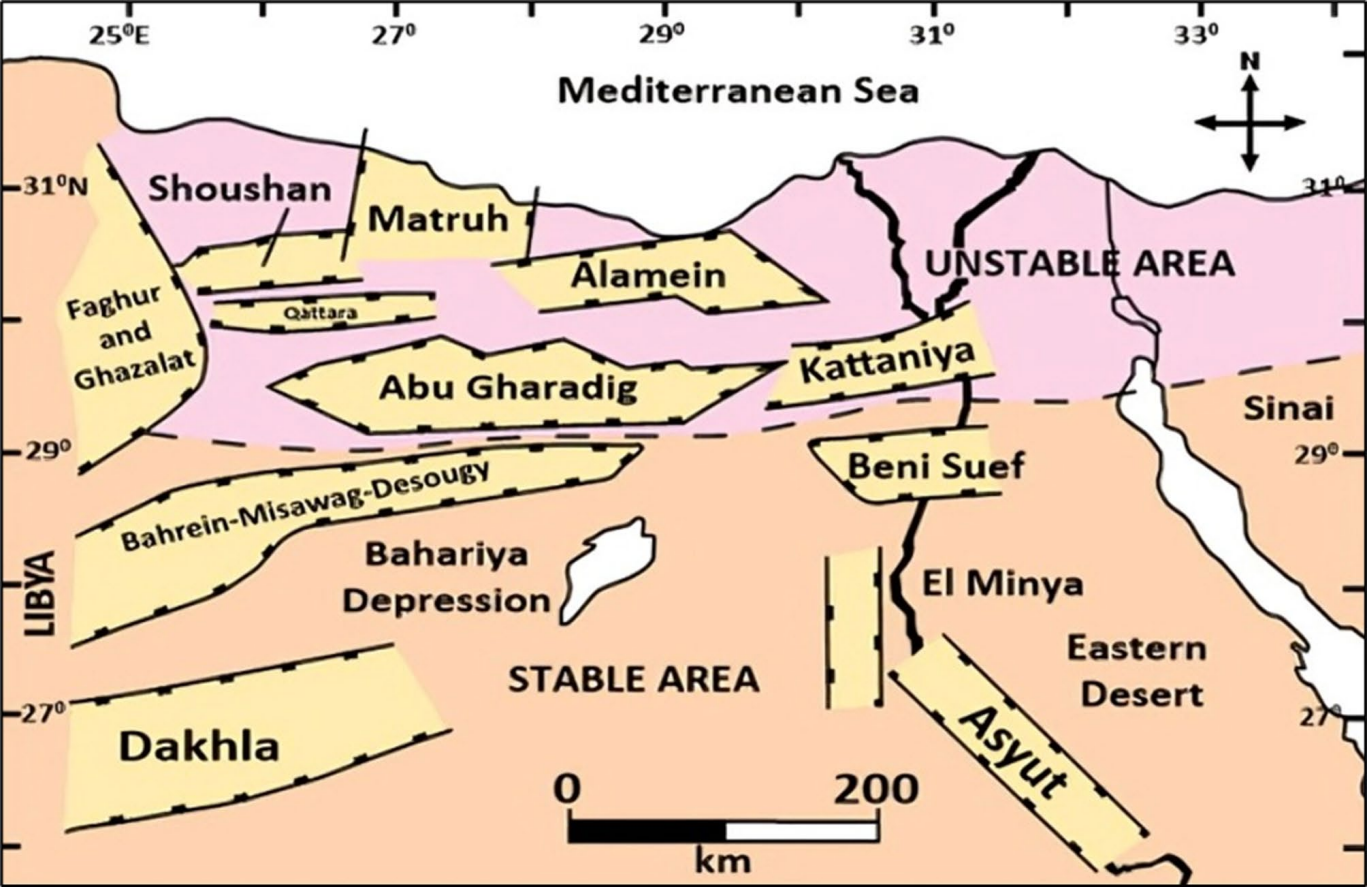
Tectonic and structural map of Egypt’s Western Desert showing regional structural trends and basin geometry, adapted from references^21^.

The tectonic evolution of the Abu Gharadig Basin has played a crucial role in shaping its reservoir architecture and trapping mechanisms (Fig. 3). The basin developed through multiple phases of tectonic activity, beginning with Late Jurassic to Early Cretaceous extensional rifting associated with the opening of the Neotethys Ocean^20,21^. This rifting generated a series of half-grabens and normal fault systems, primarily oriented ENE–WSW and E–W, which influenced sedimentation patterns and reservoir distribution. During the Late Cretaceous and Paleogene, compressional forces related to the Syrian Arc tectonic phase led to basin inversion, forming faulted anticlines and structural closures that now serve as effective hydrocarbon and potential CO₂ traps^20,21^. These structural elements also affect reservoir compartmentalization and influence CO₂ injectivity and containment, factors that are critical for safe and effective sequestration.

**Fig. 4:**
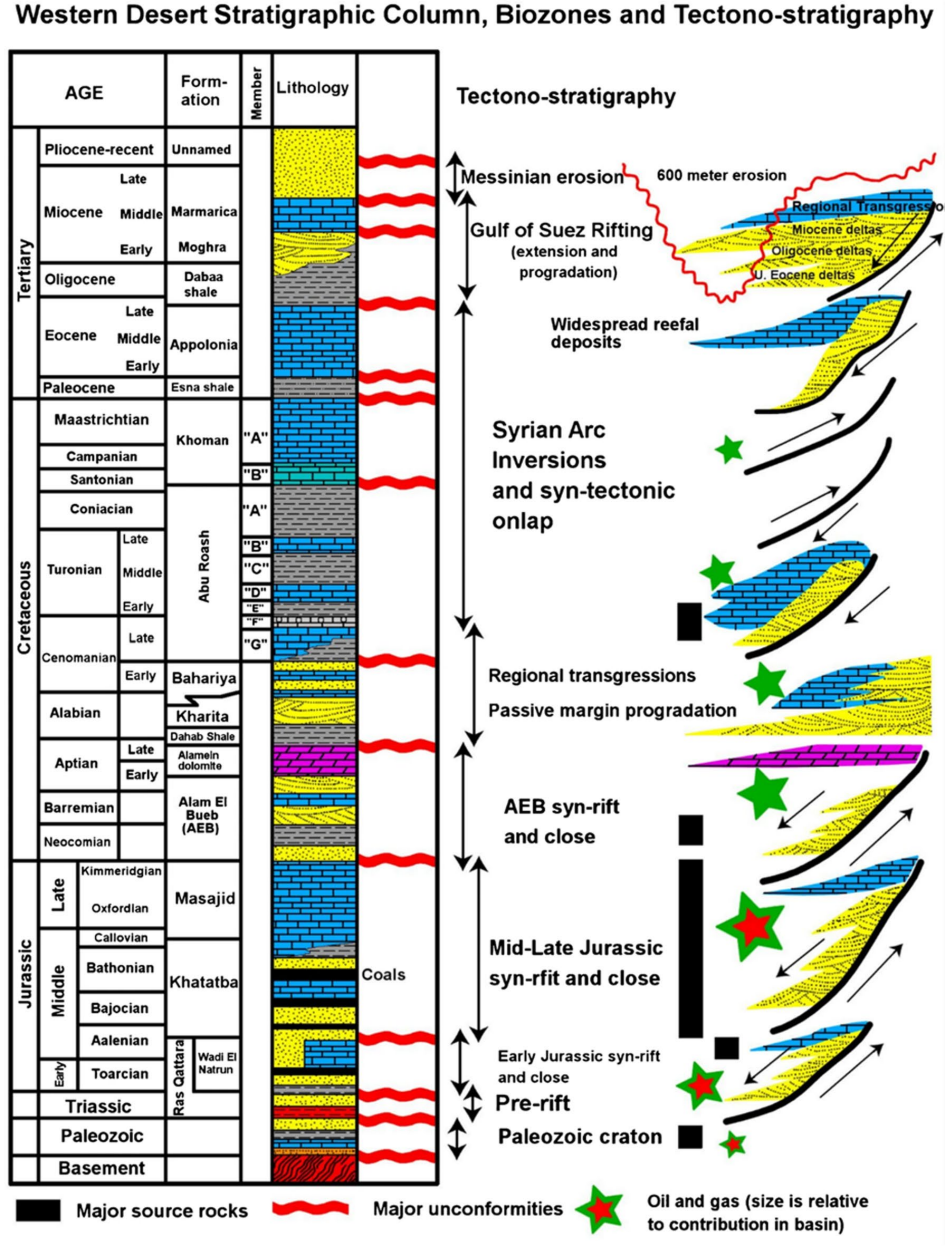
Stratigraphic framework of Egypt’s Northwestern Desert showing key lithostratigraphic units and depositional environments relevant to hydrocarbon exploration and CO₂ storage^21^.

Stratigraphically, the Western Desert comprises a complete sedimentary record from Precambrian basement through to Recent deposits (Fig. 4). The Paleozoic units are generally thin and consist of continental sandstones and shales, while the Mesozoic succession especially from the Jurassic and Cretaceous periods is dominated by marine carbonates, shales, and sandstones. This interval includes key petroleum-bearing formations such as Khatatba, Bahariya, and Abu Roash, with the latter being particularly important in the Abu Sannan Field. Overlying these Mesozoic sequences are Cenozoic deposits, mainly composed of clastic sediments from fluvial and deltaic systems. The well-established stratigraphic framework, supported by extensive seismic and well data, facilitates detailed geological modeling and reservoir analysis, both for hydrocarbon development and CO₂ storage assessment^22^.

The Abu Roash Formation, particularly the D Member, is of special interest in the context of CO₂ sequestration. The Abu Roash D Member consists of fractured carbonate rocks that have developed relatively high porosity due to natural fracturing, enhancing both fluid flow and storage capacity. In the Abu Sannan Field, this unit has been extensively exploited for oil production and is now largely depleted, presenting a promising target for CO₂ injection. Its favorable reservoir characteristics, including good porosity, moderate permeability, and well-defined structural traps, make it well-suited for long-term CO₂ storage^23–25^. Moreover, the presence of overlying shale-rich units such as the Abu Roash E and F Members provides effective sealing, critical for ensuring the integrity and security of the storage site. The integration of petrophysical analysis, seismic interpretation, and reservoir modeling supports the potential of Abu Roash D as a reliable and efficient CO₂ storage reservoir in the Western Desert^26,27^.

## Methodology

**Fig. 5:**
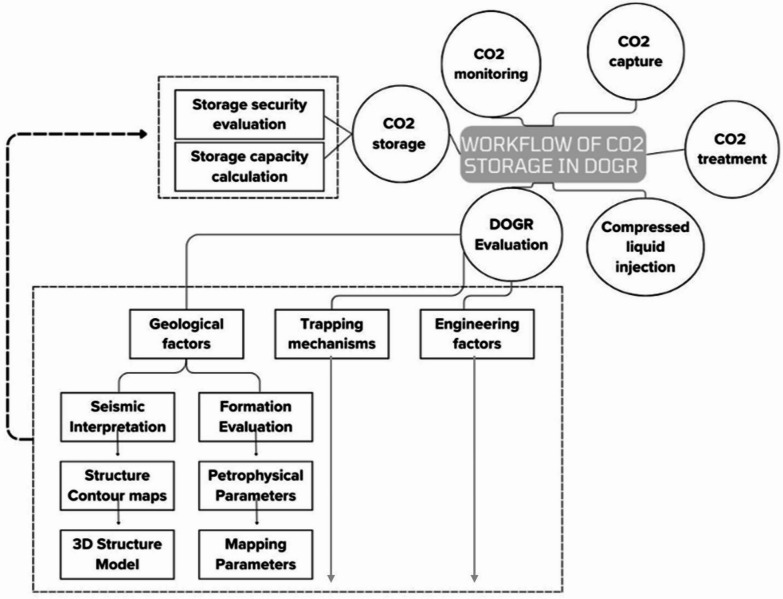
Methodological framework for carbon capture and storage assessment.

CCS represents a multi-stage process comprising the capture of CO₂ from major anthropogenic sources, purification and compression to pipeline specifications, transportation via dedicated infrastructure, injection into suitable geological formations, long-term storage within the subsurface, and continuous monitoring to ensure containment and security^17–19^. Each stage is technically significant; however, the present study concentrates exclusively on the storage stage. Specifically, the focus is placed on evaluating DOGRs in the Abu Roash D Member of the Abu Sannan Oil Field, Western Desert, Egypt, as potential CO₂ storage sites. This emphasis reflects the dual advantage of DOGRs, which combine proven reservoir properties with existing infrastructure, thereby reducing both uncertainty and implementation costs^26–29^. Figure 5 illustrates the complete workflow of CCS; however, this article focuses specifically on storage efficiency in the DOGRs, based on geological factors, trapping mechanisms, and storage capacity calculations.

**Fig. 6:**
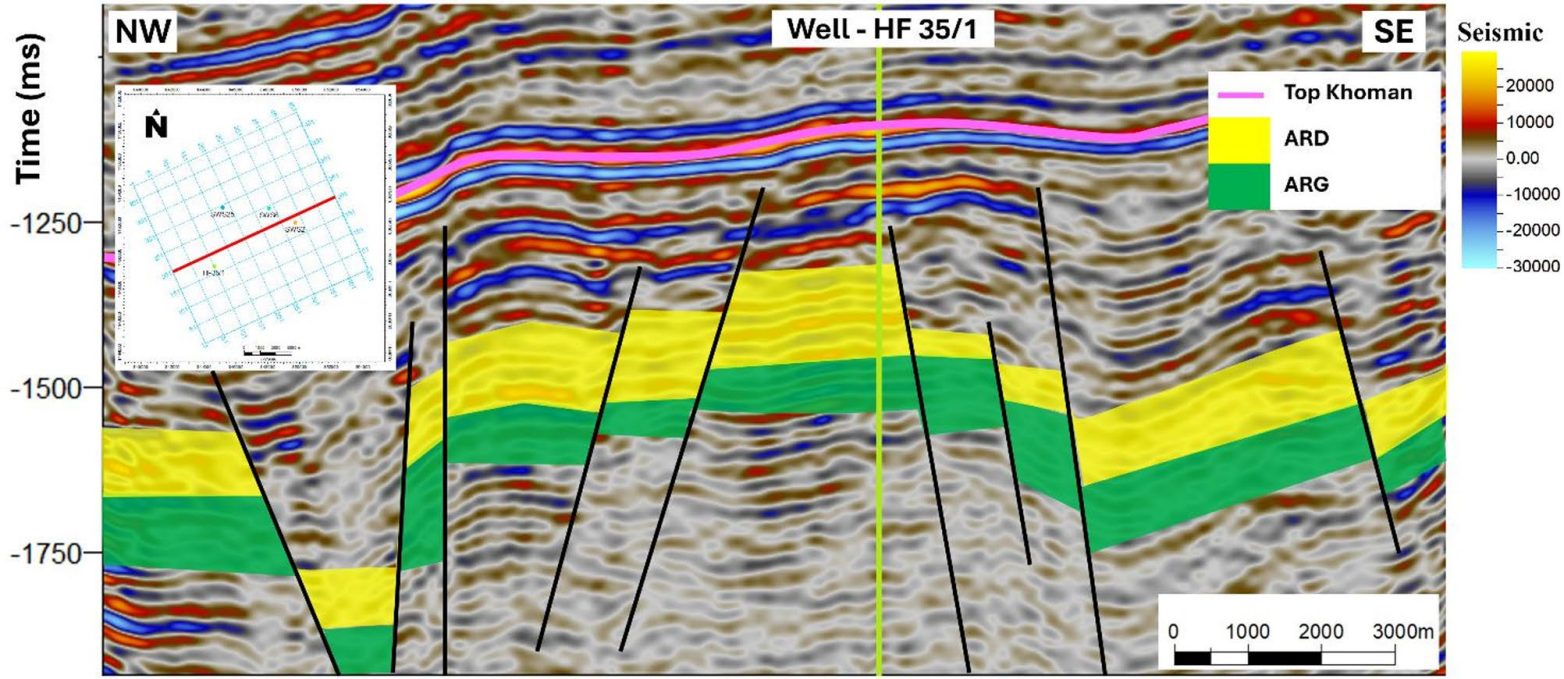
Interpreted seismic section from the southern part of the Amal area, illustrating extensional faults and significant horizons.

**Fig. 7:**
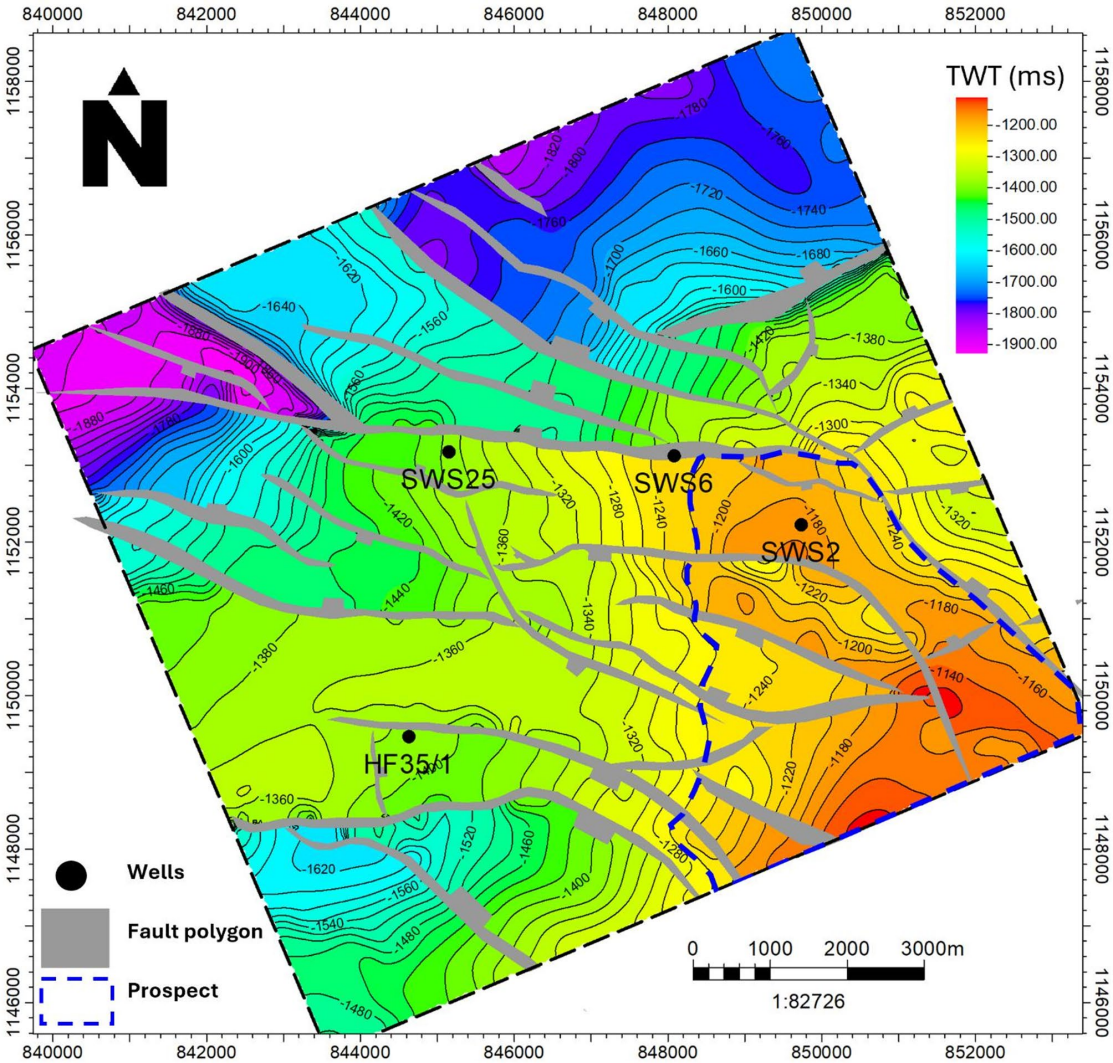
Time structure contour map of the Abu Roash D Formation with well locations and planned prospect.

**Fig. 8:**
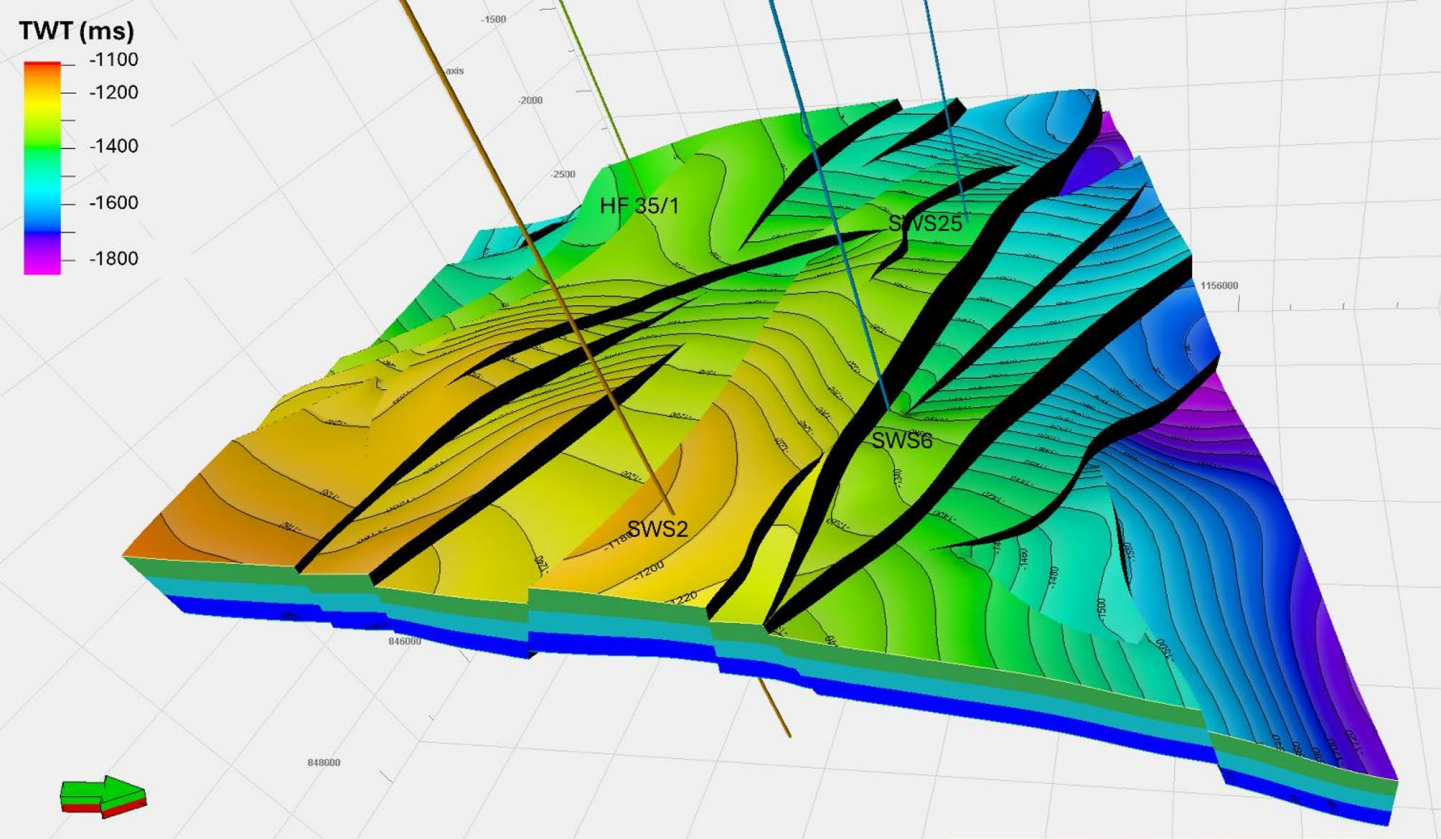
Three-dimensional structural model of the study area, with delineated zones.

The assessment of storage potential was conducted through an integrated workflow combining seismic interpretation, petrophysical analysis, and volumetric capacity estimation. The process commenced with the interpretation of 2D seismic reflection data (SEG-Y format), which had been pre-processed in the time domain and integrated with available well log datasets. Seismic horizons were systematically picked to establish the stratigraphic framework, while fault mapping delineated structural compartmentalization and potential migration pathways. These interpretations (Fig. 6) were used to generate 2D structural contour maps of the Abu Roash D Member (Fig. 7), which subsequently informed the construction of 3D geostatic models (Fig. 8). These models provided a detailed representation of reservoir architecture and connectivity, allowing volumetric space available for CO₂ injection to be accurately constrained^28,30–33^.

**Fig. 9:**
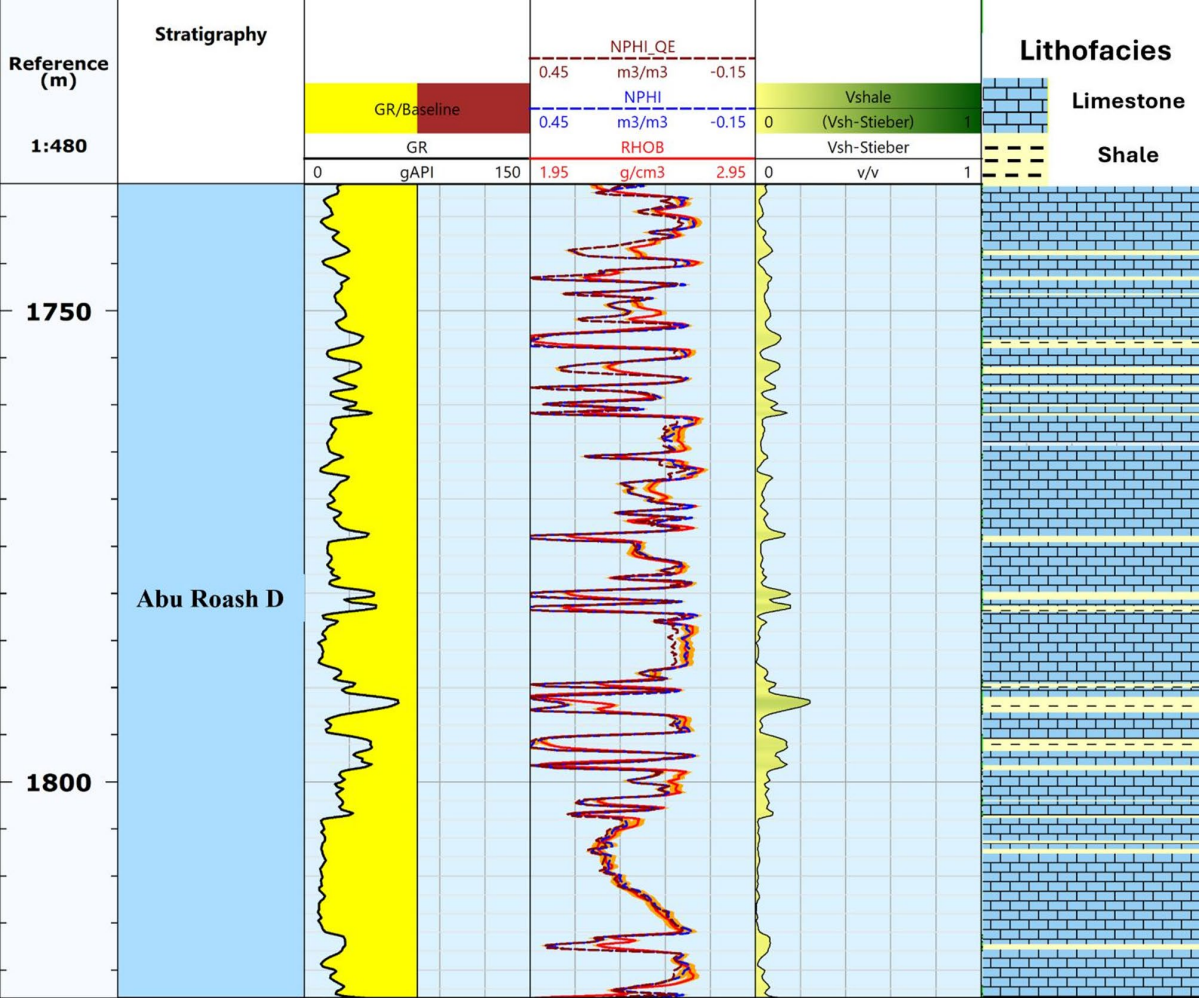
Example illustrating the Abu Roash D zone as an area of interest characterized by low gamma-ray values and high porosity.

**Fig. 10:**
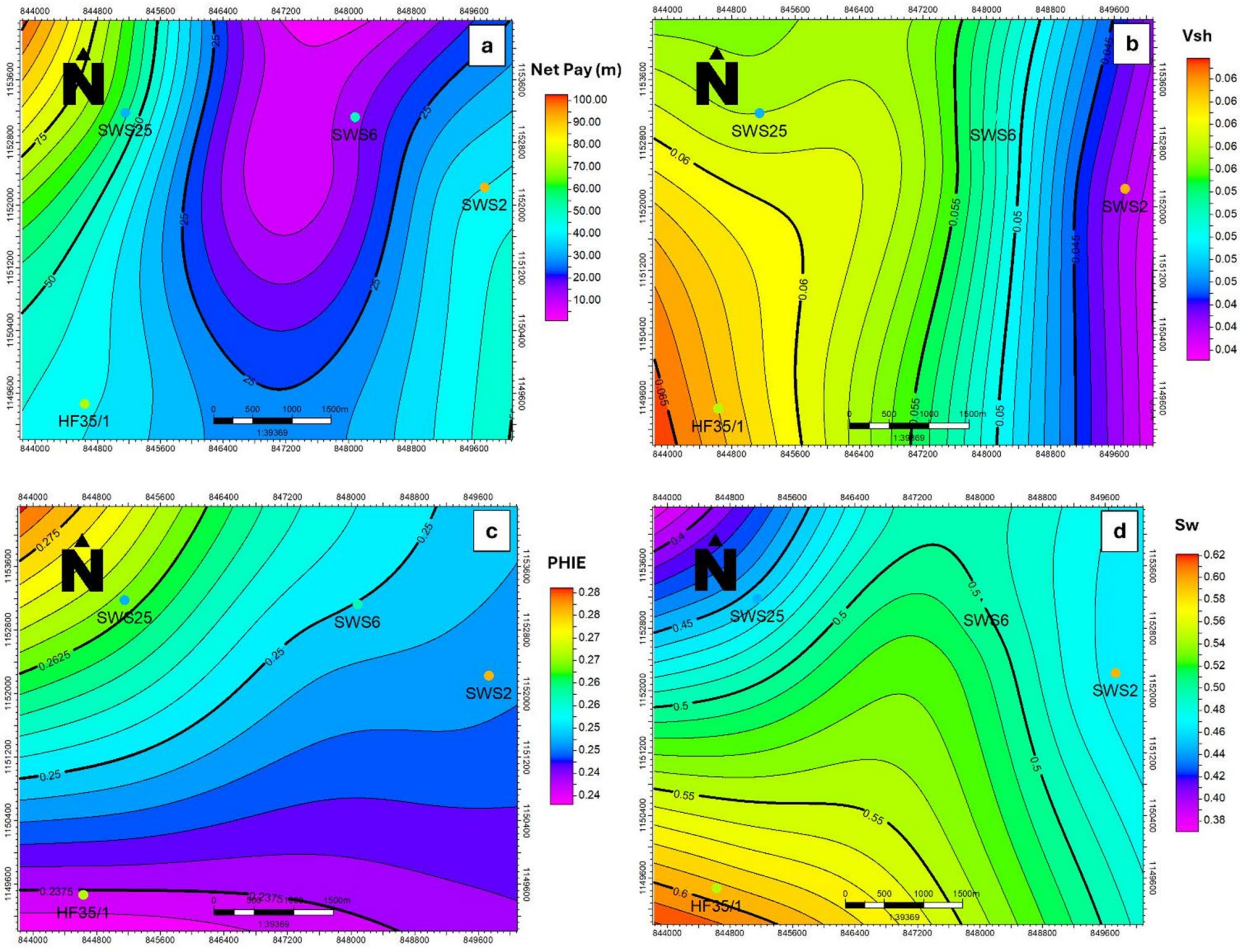
The Abu Roash D Formation properties: (**a**) thickness; (**b**) shale volume; (**c**) effective porosity; (**d**) water saturation.

Petrophysical evaluation was then performed using a suite of wireline logs (Fig. 9), including gamma ray, density, neutron, sonic, and resistivity. From these datasets, key reservoir parameters were derived: shale volume, porosity (total and effective), water saturation (Sw), and permeability. These properties were laterally mapped to a 2D property maps (Fig. 10). Such integration ensured that the heterogeneity of the reservoir, which strongly influences injectivity and storage efficiency, was appropriately captured^34–36^.

CO₂ storage capacity was estimated using static volumetric methods, incorporating pore volume, irreducible water saturation, CO₂ density, and the formation volume factor. This approach, consistent with established methodologies and published eqn.s, provides first-order estimates of storage potential^18,19,29^. While dynamic simulations and coupled geomechanical modeling were beyond the scope of the present study, their application in future work is recommended to refine predictions of CO₂ plume migration, pressure evolution, and cap rock integrity^14,37–39^.

The methodology presented here therefore establishes a rigorous framework for assessing DOGRs as CO₂ storage sites, linking seismic interpretation, petrophysical characterization, and volumetric analysis. The results provide the foundation for the subsequent evaluation of storage potential within the Abu Roash D Member, highlighting both opportunities and limitations of depleted hydrocarbon reservoirs for long-term carbon sequestration in the Western Desert of Egypt.

In summary, the adopted workflow integrates seismic interpretation, petrophysical evaluation, and volumetric estimation to accurately assess the CO₂ storage capacity within the Abu Roash D Member. To ensure the robustness of this approach, the following section presents a detailed description of the datasets utilized, including the seismic and well log data, along with the software platforms employed for data integration and analysis. This provides the foundation for the subsequent interpretation and modeling stages of the study.

### Dataset

The research employed 2D seismic profile data in the time domain, provided in SEG-Y format, along with a set of in-lines, crosslines, and comprehensive wireline log data from four wells (SWS25, SWS6, SWS2 and HF35/1). The wireline logs included key measurements such as GR, Density, Neutron Porosity, Sonic, and Resistivity. These seismic and well log datasets were integrated using Petrel software to develop a three-dimensional structural model of the study area. In parallel, petrophysical analyses were performed using Techlog software to evaluate reservoir properties and support the characterization of potential CO₂ storage intervals.

### Methodological framework

All available data were integrated to construct detailed 3D structural models of the Abu Roash D Member. Following the development of these static models to get the prospective trap volume, petrophysical analysis were employed to map and track the key petrophysical properties, including shale volume, total and effective porosity, water saturation, and permeability. The resulting model and maps provided a comprehensive representation of the reservoir and were subsequently used to assess and estimate the CO₂ storage potential within the Abu Roash D interval^29,30^.

#### 3D geo-static modeling

This study focused on the Abu Roash reservoir, particularly due to its favorable reservoir characteristics and depositional facies, which make it a suitable candidate for CO₂ storage. The modeling workflow began with a well-to-seismic tie, which represents the first step of seismic interpretation. As shown in Fig. 6, well HF35/1 was converted to the time domain using check-shot velocity data to identify the formation tops on the seismic time section, with a projection distance of approximately 500 m. The same procedure was applied to the other wells as part of the interpretation process, then the key seismic horizons were identified and tracked laterally to delineate subsurface structures, stratigraphic features, and the overall geometry of the reservoir. Faults and associated structural elements were mapped in detail to define potential traps, assess their spatial extent, and estimate their volumetric capacity based on (Eq. 6). Based on this interpretation, 2D structural contour maps of the target reservoir intervals were constructed to support subsequent modeling efforts^29–31^.

Building on the results of seismic interpretation, the three-dimensional modeling of CO₂ storage began with the integration of interpreted structural surfaces and fault data. These 2D outputs were converted into time-based 3D surfaces, allowing for an accurate representation of subsurface structural trends and the identification of potential CO₂ storage sites. The modeling process continued with the creation of a 3D fault framework, gridding of the reservoir volume, and generation of geological horizons. Faults provided the structural framework necessary to guide the construction of the 3D grid, while seismic horizons defined the vertical layering, ensuring that the model accurately reflected the stratigraphic architecture of the Abu Roash reservoir^32,33^.

The finalized structural model incorporated all interpreted geological elements, including horizons and faults, and accurately defined the contacts between them. This ensured that the model realistically represented the geometry of the reservoir, fault connectivity, and horizon relationships. To enhance the model’s resolution and improve CO₂ storage estimation, the reservoir was further subdivided into zones based on its stratigraphic framework, with fine-scale layering applied to capture key flow units and heterogeneities. This comprehensive 3D structural model provided the foundation for evaluating the CO₂ storage potential in the Abu Roash reservoir, enabling a robust analysis of storage capacity, injectivity, and containment security. The detailed representation of subsurface features achieved through this modeling approach is critical for ensuring accurate resource assessment and the effective planning of future CO₂ sequestration efforts^34,35^.

#### Petrophysical analysis

Reservoir property maps were developed using key output parameters derived from petrophysical evaluation, including net pay thickness, shale volume, total and effective porosity, as well as water saturation. Facies classification relied on the analysis of wireline log data. Initially, GR logs were employed to distinguish between shale and non-shale intervals. Subsequently, lithological characterization of the non-shale sections was performed using additional log data, such as Neutron, Density, and Sonic logs^36^. All petrophysical parameters of the Abu Roash D Formation were calculated using standard formulas for four wells (SWS25, SWS6, SWS2, and HF35/1) and subsequently interpolated and contoured using the kriging algorithm in Techlog software to generate property maps that depict the lateral variability.

Shale volume (Vsh) is a crucial factor in determining both porosity and permeability, which directly impacts reservoir quality. Accurate estimation of Vsh is therefore essential. In this study, the GR method was utilized to compute Vsh (Eq. 1). High GR readings generally indicate shaly formations due to the presence of radioactive minerals such as potassium-bearing clays, while low GR values are typically associated with clean sandstones or carbonates, and the resulting values were spatially mapped to assess lateral variations across the field. Total porosity, a key measure of a reservoir rock’s storage capacity, was estimated using a combination of density and neutron log data (Eq. 2), which provided reliable estimates specific to the reservoir conditions. Effective porosity, which influences the estimation of permeability and fluid flow potential, was calculated by incorporating neutron-density porosity, Vsh, and shale porosity using a standard eqn. (Eq. 3)^37,38^.

Water saturation (Sw), representing the proportion of pore space filled with water, was estimated using models suited to the lithological context. Among various methods, Archie’s Eqn. is generally applied to clean sandstones, while formations with mixed lithologies or higher clay content require alternative models. Given the presence of thin shale interbeds in the target formations, the Indonesian formula (Eq. 4) was selected for its suitability in handling such complex lithologies. This method adjusts for the effect of shale on Sw calculations and improves the accuracy of water saturation estimations in “dirty” formations^39^.

Permeability, which describes the connectivity of pore spaces and the ease with which fluids can move through the rock, was estimated in the absence of core data by applying an empirical method. The Wyllie-Rose approach (Eq. 5), widely recognized for its application in data-limited environments, was employed to compute permeability values. This methodology enabled a comprehensive understanding of reservoir performance based on available log data.

1 $$\:Vsh\:=\frac{{GR}_{log}-\:{GR}_{min}}{{GR}_{max}\:-\:{GR}_{min}}$$

2 $$\:{\varnothing\:}_{T}=\:\frac{{\varnothing\:}_{N}+\:{\varnothing\:}_{D}\:}{2}$$

3 $$\:{\varnothing\:}_{eff}=\:{\varnothing\:}_{T}-\left(\:{V}_{sh}*\:{\varnothing\:}_{sh}\right)$$

4 $$\:\frac{1}{\sqrt{{R}_{t}}}=\left[\sqrt{\frac{{\varnothing\:}_{T}^{m}}{a{R}_{w}}}+\frac{{{V}_{sh}}^{\left(\frac{1-{V}_{sh}}{2}\right)}}{\sqrt{{R}_{cl}}}\right]{{S}_{w}}^{n}$$

5 $$\:k=\:a*\frac{{\varnothing\:}_{T}^{b}}{{Swir}^{c}}$$

In this context, $\:{R}_{t}$ refers to the true resistivity of the formation, while $\:{V}_{sh}$ indicates the volume fraction of clay or shale. The $\:{GR}_{log}$ denotes the gamma-ray log reading, with $\:{GR}_{min}$ and $\:{GR}_{max}$ representing its minimum and maximum observed values, respectively. $\:{\varnothing\:}_{T}$ corresponds to total porosity, $\:{\varnothing\:}_{N}$ to neutron-derived porosity, and $\:{\varnothing\:}_{D}$ to porosity derived from density logs. $\:{\varnothing\:}_{eff}$ indicates effective porosity, and $\:{\varnothing\:}_{sh}$ denotes the porosity attributable to shale content. The tortuosity, cementation, and saturation exponent factors are expressed as $\:a$, $\:m$ and *n* $\:\text{r}\text{e}\text{s}\text{p}\text{e}\text{c}\text{t}\text{i}\text{v}\text{e}\text{l}\text{y}.\:\:{R}_{w}$ represents the resistivity of the formation water, and $\:{R}_{cl}$ is the resistivity measured within shale zones. Permeability, denoted as $\:k$, is quantified in millidarcies (mD), Constants are defined as b = 4.5, and c = 2.

#### CO_2_ storage capacity

Assessing the potential of a reservoir for CO₂ storage requires a thorough evaluation of its petrophysical characteristics. Multiple mechanisms contribute to effective CO₂ sequestration, including structural trapping, residual (capillary) trapping, and solubility trapping. In this research, the storage capacity for CO₂ was estimated through detailed analysis using Petrel software. In this study, the storage capacity was estimated through the integration of seismic data, which defines the structural trap or prospective area capable of storing CO₂. The trap area and thickness were derived from 2D maps and the 3D static model, providing the total trap volume. This volume was then converted to pore volume by multiplying it with porosity values calculated from well data, thereby excluding the rock matrix and shale fraction. The effective pore space was further refined by accounting for water saturation, also determined from well logs, to exclude the portion occupied by formation water. The remaining pore volume, available for CO₂ storage, was subsequently multiplied by the density of CO₂, the formation volume factor, and the storage efficiency factor to determine the final storage capacity. This approach incorporates petrophysical data to quantitatively determine the reservoir’s ability to store CO₂ securely within its pore spaces. The estimation methodology follows established principles and is supported by a standard formula (Eq. 6) to ensure accuracy and consistency in capacity calculations^3^.

6 $$\:MCO2\:=\:Vpv\:\times\:\:1\:-\:Swir\:\times\:\:B\:\times\:\:\rho\:CO2\:\times\:\:E$$

where $\:MCO2$ represents CO2 storage capacity, $\:Swir$ stands for irreducible water saturation, and $\:Vpv$ signifies the total pore volume in cubic meters (m^2^). Additional constants include $\:a$ = 10,000.0, $\:b$ = 4.5, and $\:c$ = 2. The formation volume factor is labeled as $\:B$, $\:\rho\:CO2$ is the density of carbon dioxide, and $\:E$ represents the efficiency factor for CO₂ storage.

Following the calculation of the key petrophysical parameters, these values were spatially mapped across the study area to visualize lateral variations, which play a critical role in influencing the distribution and capacity of CO₂ storage.

## Results and discussion

### Structural contour maps

The interpretation of seismic data began with examining seismic reflectors and structural features in the time domain, allowing the construction of detailed maps that illustrate subsurface structures and reservoir configurations. This process was essential for identifying subsurface traps capable of holding fluids, tracing their lateral extent, and estimating their potential volumes. By accurately mapping these features, the interpretation provided key insights into the distribution of reserves, which are vital for exploration and development activities in the area. All available seismic lines were interpreted to delineate the top and bottom of the Abu Roash D Formation laterally across the field. The top horizon is marked by a trough that reflects the transition from carbonate to sandstone. Similarly, the bottom horizon, corresponding to the Abu Roash G Member, is also characterized by a trough. In addition, faults were systematically identified and tracked, reveining that the area is affected by extensional tectonics expressed through normal faulting. These tectonic processes have led to the development of normal-faulted structural styles, including rotated fault blocks, horsts, and grabens (Fig. 6).

Following the tracking of the Abu Roash D reflector and fault network, a structural contour map was constructed to analyze the lateral distribution of the subsurface structures across the area (Fig. 7). The map indicates that the region is strongly influenced by a system of ENE–WSW and NW–SE trending normal faults, which have produced rotated fault blocks, horsts, and grabens. The mapped horizon ranges in depth from about 1200 ms to 1900 ms, with shallower structural highs observed in the southeastern part of the area. These structural settings not only define the tectonic framework but also create favorable trap geometries, making the Abu Roash D Formation a promising target for CO₂ storage (Fig. 7).

### 3D Geo-static model

The assessment of CO₂ storage capacity in the Abu Sannan Oil Field requires a comprehensive three-dimensional (3D) structural modeling approach to accurately evaluate the present subsurface architecture. This process allows detailed visualization of the subsurface by generating cross-sections in multiple directions, enabling a more precise assessment of the heterogeneous reservoirs. The structural modeling workflow was conducted in successive phases, each building upon the outcomes of seismic interpretation, including well tops, thickness maps, interpreted horizons, and identified faults. The first step involved defining the fundamental structural geometry from seismic data to ensure a reliable representation of the subsurface. Next, a fault framework was constructed from the interpreted fault network, establishing the structural skeleton of the reservoir. Finally, the Abu Roash D horizon was modeled with a detailed definition of the relationships between faults and their structural influence on the reservoir. By integrating these stages, the 3D structural model provides a robust and dynamic representation of the subsurface, enhancing the evaluation of CO₂ storage potential and improving the understanding of reservoir behavior.

The Abu Roash D model shows that the field is dominated by a series of normal-faulted structural styles, including tilted fault blocks, half-grabens, grabens, and horsts, trending ENE–WSW and NW–SE. These structures display variable throws to the northeast and southwest, creating significant displacement across the field (Fig. 8). Such tectonic variability results in well-defined structural traps that are highly suitable for CO₂ storage. The model also indicates that the thickness of the Abu Roash D Formation increases toward the southern part of the field.

Following the construction of the static model, it was essential to capture the internal reservoir architecture and flow heterogeneity. To achieve this, the Abu Roash D zone was subdivided into layers, enabling a more accurate representation of vertical and lateral variations in flow units. The model highlights that the southeastern structural highs, corresponding to faulted blocks, represent particularly favorable sites for CO₂ storage. Additionally, the integration of the detailed fault framework with fine grid resolution improves the precision of storage capacity estimates and ensures a better understanding of reservoir performance.

### Petrophysical analysis

Petrophysical analysis is a fundamental step in evaluating the potential of reservoir units for CO₂ storage, as it provides essential insights into formation quality, storage capacity, and overall suitability. The process begins with the identification of key zones of interest (Fig. 9) through detailed analysis of log signatures, which allows for the delineation of distinct lithological units and their petrophysical characteristics. Within the Abu Roash D Formation, this approach enabled the selection of intervals with favorable reservoir properties for further assessment.

A comprehensive evaluation of facies distribution, porosity (ɸ), permeability (k), shale volume (Vsh), and Sw was then conducted to define the reservoir performance and its implications for efficient CO₂ storage. The results indicate that the Abu Roash D contains intervals with excellent porosity and permeability, coupled with relatively low water saturation. These characteristics enhance both the storage capacity and sealing efficiency of the formation, ensuring that it can securely accommodate and retain injected CO₂ over geological timescales.

To refine the spatial understanding of reservoir quality, petrophysical maps of facies distribution, shale volume, water saturation, effective porosity, and permeability were generated. These maps provide a clear visualization of the lateral variations within the Abu Roash D Formation, offering valuable insights into reservoir heterogeneity and its suitability for long-term CO₂ storage. Such detailed petrophysical characterization is essential for identifying the most favorable zones, evaluating storage capacity, and assessing the efficiency of CO₂ sequestration while reducing potential risks of leakage.

The facies analysis of the Abu Roash D Formation shows that it is composed predominantly of fractured carbonates with thin shale streaks distributed across the area. Interpretation of wireline logs confirms that carbonate intervals dominate, providing favorable storage capacity and injectivity, while the limited shale interbeds act as minor baffles without significantly reducing reservoir connectivity. The thickness of the Abu Roash D Formation ranges between 10 m and 100 m (Fig. 10a), with the maximum thickness observed in the northwestern part of the region. In addition, the Khoman Formation provides a regionally extensive sealing unit of considerable thickness, composed mainly of impermeable chalk. This formation extends across a wide area, not limited to the Abu Sannan field, and offers an excellent cap rock that ensures long-term containment and minimizes the risk of CO₂ migration. The combination of fractured carbonate reservoir facies within the Abu Roash D and the regional Khoman seal highlights the strong potential of the Abu Roash D Formation for secure and efficient CO₂ storage.

The shale volume (Vsh) within the Abu Roash D Formation was calculated from GR logs, and the results confirm that it is a clean and relatively homogeneous reservoir, with only a very low proportion of shale. Vsh values range between 0.04 and 0.06, emphasizing the overall clean character of the formation. Slightly higher values are observed in the southwestern part of the area, but even these remain very low and have no significant impact on reservoir quality (Fig. 10b).

This consistently low shale content is particularly advantageous for CO₂ storage, as it indicates a predominance of clean fractured carbonates with minimal clay-related pore blocking. The lack of significant shale ensures better pore connectivity, higher effective porosity, and enhanced injectivity, which together improve the ability of the formation to accommodate large volumes of injected CO₂. Furthermore, the homogeneity of the Abu Roash D reduces reservoir heterogeneity-related risks, ensuring more predictable plume migration and more efficient utilization of available pore space. These factors underline the strong suitability of the Abu Roash D Formation as a secure and effective CO₂ storage reservoir.

The effective porosity of the Abu Roash D Formation was calculated using the standard petrophysical eqn. based on density, neutron porosity logs and shale volume, as previously outlined. The results show remarkably high values, ranging between 0.24 and 0.28 (Fig. 10c), with the maximum observed toward the northwestern part of the study area. These values are exceptionally good for carbonate reservoirs and reflect the outstanding reservoir quality of this unit. The very low shale content, confirmed by the Gamma Ray-derived shale volume (Vsh), further enhances these porosity levels by minimizing pore obstruction and preserving pore connectivity within the carbonate matrix. Such high effective porosity, coupled with the clean and homogeneous character of the formation, provides a substantial pore volume that is highly favorable for CO₂ storage. The abundance of interconnected pores ensures excellent injectivity and large storage capacity, while the negligible shale streaks reduce the likelihood of compartmentalization. These combined characteristics highlight the Abu Roash D Formation as a prime and reliable target for long-term CO₂ sequestration.

The water saturation (Sw) within the Abu Roash D Formation ranges from 0.38 to 0.62 (Fig. 10d), with the highest values concentrated in the southwestern part of the study area. These values indicate that a significant portion of the reservoir pore space is not fully saturated with formation water, meaning hydrocarbons have already been produced from this zone. Such a distribution reflects a reservoir moving toward depletion, which is a key indicator of remaining storage capacity and injection potential. This condition presents a strong opportunity for CO₂ injection and enhanced oil recovery (EOR). The depleted state of the reservoir, combined with its relatively low water saturation, creates additional pore volume that can be effectively filled with injected CO₂. This not only allows for improved hydrocarbon recovery but also ensures that injected CO₂ can be securely stored within the available pore network. Therefore, the observed Sw values confirm that the Abu Roash D Formation is highly favorable for dual utilization boosting recovery efficiency while simultaneously serving as a long-term CO₂ storage site.

Permeability within the Abu Roash D Formation is generally high, largely due to its fractured carbonate nature, which creates extensive interconnected flow pathways. Permeability values in such fractured systems typically range from tens to several hundred millidarcies (mD), with localized fracture corridors potentially reaching even higher values. These enhanced permeability characteristics ensure excellent reservoir performance, supporting both hydrocarbon production and efficient CO₂ injection. When coupled with the unit’s high porosity and very low shale content, the Abu Roash D Formation stands out as a highly suitable candidate for secure and long-term CO₂ storage.

### CO_2_ storage potential

The southeastern part of the Abu Roash D Formation represents the most favorable target for CO₂ storage, as it occupies the highest structural part within the study area, ensuring effective up dip containment and minimizing the risk of leakage. Petrophysical evaluation further supports this conclusion, as this sector is characterized by excellent reservoir properties, including high effective porosity, uniform permeability enhanced by fractures, and very low shale content. These combined factors make the southeastern area the most promising site for secure and efficient CO₂ sequestration. In contrast, other parts of the reservoir display lower storage potential due to reduced porosity and greater shale heterogeneity.

The Abu Roash D reservoir has an estimated CO₂ storage capacity of 72,657 Mt using (Eq. 6), supported by its high porosity and favorable permeability that allow efficient injection and long-term containment. These characteristics establish it as an excellent candidate for enhanced oil recovery and geological sequestration in the Western desert. Table 1 presents the of pore volume and its corresponding CO₂ storage potential, highlighting the relationship between pore volume size and storage capacity.

**Table 1:** Pore volume and corresponding CO₂ storage potential, illustrating the relationship between pore volume size and storage capacity.

	Pro volume ( x10^6^ m^3^)	CO2 storage potential (Mt)
Prospect	448	72,657

## Conclusion

This study evaluated the CO₂ storage potential of the Abu Roash D reservoir in the Abu Sannan Oil Field, Western Desert, Egypt. The assessment focused on reservoir quality, structural configuration, and storage capacity by integrating seismic interpretation, petrophysical analysis, and volumetric estimation. The results provide new insights into the suitability of depleted oil and gas reservoirs (DOGRs) for secure and efficient carbon storage. The main conclusions are as follows:

1. The Abu Roash D reservoir demonstrates excellent suitability for CO₂ sequestration, supported by its high porosity (~ 0.25), low shale content (~ 0.05), moderate water saturation (~ 0.40), and relatively uniform permeability in the tens of millidarcies.
2. Structural interpretation reveals a network of normal faults forming tilted blocks, horsts, and grabens that provide effective closures and favorable traps for long-term CO₂ containment.
3. The southeastern portion of the reservoir represents the most promising storage site, combining superior reservoir quality with advantageous structural positioning.
4. Capacity assessment indicates that the Abu Roash D reservoir can securely store approximately 72,657 Mt of CO₂, positioning it as a strategic site for large-scale carbon storage in Egypt.
5. The presence of existing oil and gas infrastructure, including wells, pipelines, and surface facilities, enables cost-effective CCS implementation with minimal additional investment.
6. Advanced monitoring technologies such as 3D seismic surveys, well logging, and production data analysis can ensure effective plume tracking and secure long-term storage performance.

The methodology applied in this study provides a transferable framework for evaluating CO₂ storage potential in other reservoirs of Egypt’s Western Desert, thereby supporting the country’s carbon management strategy and aligning with global climate mitigation objectives. Future research should incorporate uncertainty analysis, dynamic reservoir simulation, and coupled geomechanical modeling to refine storage capacity estimates, assess potential risks, and enhance predictive reliability for safe and large-scale CCS deployment.

## Data Availability

The corresponding author has to be contacted in case of any queries or requirement of data.
